# High-Quality Draft Genome Sequences of Eight Bacteria Isolated from Fungus Gardens Grown by Trachymyrmex septentrionalis Ants

**DOI:** 10.1128/MRA.00871-18

**Published:** 2018-07-19

**Authors:** Sarah Kopac, Hannah Beatty, Philip Gialopsos, Marcel Huntemann, Alicia Clum, Alexander Spunde, Manoj Pillay, Krishnaveni Palaniappan, Neha Varghese, Natalia Mikhailova, Dimitrios Stamatis, T. B. K. Reddy, Chris Daum, Vivian Ng, Natalia Ivanova, Nikos Kyrpides, Tanja Woyke, Jonathan L. Klassen

**Affiliations:** a Department of Molecular and Cell Biology, University of Connecticut, Storrs, Connecticut, USA; b Department of Energy Joint Genome Institute (DOE JGI), Walnut Creek, California, USA; University of Maryland School of Medicine

## Abstract

For their food source, Trachymyrmex septentrionalis ants raise symbiotic fungus gardens that contain bacteria whose functions are poorly understood. Here, we report the genome sequences of eight bacteria isolated from these fungus gardens to better describe the ecology of these strains and their potential to produce secondary metabolites in this niche.

## ANNOUNCEMENT

Fungus-growing ants (tribe *Attini*) form symbioses with a cultivar fungus belonging to the genus Leucoagaricus, which they grow in underground fungus gardens as their essential food source (1). Other bacteria also inhabit these fungus gardens and provide nutrients to the cultivar fungus, at least in some cases (2–5). These bacteria have the genetic potential to produce secondary metabolites that may mediate interspecific interactions in fungus gardens, although this remains poorly understood (6).

Trachymyrmex septentrionalis is the northernmost fungus-growing ant and occurs throughout the eastern United States (7). Its colonies are relatively small (∼1,000 ants/colony) and subsist largely on caterpillar frass, oak catkins, and some fresh plant material (8). The T. septentrionalis fungus garden microbiome remains poorly characterized (9). We therefore isolated several bacteria from T. septentrionalis fungus gardens and sequenced their genomes to better understand their potential functions within this symbiotic niche.

T. septentrionalis fungus gardens were collected in Florida, New Jersey, and North Carolina following established protocols (10). Fungus garden fragments were resuspended in phosphate-buffered saline (137 mM NaCl, 2.7 mM KCl, 10 mM Na_2_HPO_4_, and 1.8 mM KH_2_PO_4_), and bacteria were isolated on tryptic soy agar (Difco; adjusted to pH 6) using the spread plate technique. Genomic DNA was extracted from each isolate, and their 16S rRNA genes were PCR amplified as described previously (11). PCR amplicons were Sanger sequenced at the University of Connecticut DNA Biotechnology Center, and the resulting sequences were compared to the NCBI nonredundant database (12) to identify each strain.

Genomes from eight T. septentrionalis fungus garden bacteria were sequenced at the Department of Energy Joint Genome Institute (JGI). Pacific Biosciences (PacBio) SMRTbell libraries were constructed following the manufacturer’s protocol and sequenced using a PacBio RS instrument. The resulting reads were assembled using the HGAP pipeline version 2.3.0_p5. Genes were predicted using Prodigal (13) and GenePRIMP (14) and annotated using the UniProt (15), TIGRFAMs (16), Pfam (17), KEGG (18), COG (19), InterPro (20), and IMG nonredundant (21) databases. Noncoding RNAs were annotated using tRNAScanSE (22), INFERNAL (23), and the IMG's rRNA gene models (15). Additional gene prediction and annotation were performed using the IMG's ER platform (24).

The sequenced bacteria belong to the genera Bacillus, Burkholderia, Pantoea, and Serratia and poorly resolved taxa within the families Enterobacteriaceae and Micrococcaceae (Table 1). The genome of Serratia sp. JKS000199 was assembled into a single contig and is therefore complete. All other genomes were assembled into 2 to 6 contigs and are therefore high-quality drafts. These bacteria likely include both persistent and transient colonists of T. septentrionalis fungus gardens. The genome sequences of these strains will inform future studies of their ecology in T. septentrionalis symbiosis and how secondary metabolites might mediate interspecific interactions within this niche.

**TABLE 1:** GenBank accession numbers and metadata for the strains sequenced in this study

Strain name	Collection location	Collection date (day/mo/yr)	Genome size (Mb)	Coverage (×)	No. of contigs	GenBank accession no.
Serratia sp. JKS000199	Wekiwa Springs State Park, FL, USA	20/5/13	5.12	178	1	LT907843
Enterobacteriaceae sp. JKS000233	Wekiwa Springs State Park, FL, USA	20/5/13	5.46	117	6	PEES00000000
Enterobacteriaceae sp. JKS000234	Wekiwa Springs State Park, FL, USA	20/5/13	5.45	70	5	OCMY00000000
Pantoea sp. JKS000250	Wekiwa Springs State Park, FL, USA	20/5/13	4.87	90	3	QICZ00000000
Serratia sp. JKS000296	Wekiwa Springs State Park, FL, USA	20/5/13	5.15	93	4	OCMX00000000
Burkholderia sp. JKS000303	Wharton State Forest, NJ, USA	25/6/14	8.16	99	6	PDBZ00000000
Bacillus sp. JKS001846	Singletary Lake State Park, NC, USA	10/6/15	5.96	217	2	FWYG00000000
Micrococcaceae sp. JKS001869	Paynes Creek Historic State Park, FL, USA	16/11/14	2.59	239	2	PDBY00000000

### Data availability.

The whole-genome shotgun projects for strains JKS000199, JKS000233, JKS000234, JKS000250, JKS000296, JKS000303, JKS001846, and JKS001869 have been deposited in DDBJ/EMBL/GenBank under the accession numbers listed in Table 1.
